# Effect of targeted temperature management on neurological and survival outcomes in patients undergoing extracorporeal cardiopulmonary resuscitation

**DOI:** 10.1371/journal.pone.0342473

**Published:** 2026-02-10

**Authors:** Chi Chan Lee, Yu-Yu Tu, Ping-Yi Lin, You-Cian Lin

**Affiliations:** 1 Department of Pulmonary and Critical Care Medicine, University of Utah, Salt Lake City, Utah, United States of America; 2 Department of Nursing, Hungkuang University, Taichung, Taiwan; 3 Cardiovascular Surgery, Department of Surgery, China Medical University Hospital, Taichung, Taiwan; 4 Department of Medical Research, China Medical University Hospital, Taichung, Taiwan; 5 School of Medicine, China Medical University, Taichung, Taiwan; Mount Sinai Hospital, UNITED STATES OF AMERICA

## Abstract

**Objectives:**

Targeted temperature management (TTM) has been a fundamental component of post-resuscitation care for patients experiencing cardiac arrest for many years. This study aims to investigate the association between TTM and clinical outcomes in patients undergoing extracorporeal cardiopulmonary resuscitation (ECPR).

**Methods:**

This retrospective cohort study was conducted at a single tertiary medical center in Taiwan. A total of 360 patients were screened between January 2015 and May 2023, with 148 excluded. After selection, 212 patients were included in the final analysis, with 79 receiving TTM and 133 not receiving TTM.

**Findings:**

Patients in the TTM group had a higher rate of successful weaning from extracorporeal membrane oxygenation (64.6% vs. 42.1%, p = 0.002), survival to discharge (58.2% vs. 38.3%, p = 0.005) and lower cerebral performance category (CPC) scores at discharge (3.6 vs. 4.2, p = 0.001). A higher proportion of patients in the TTM group had a CPC score of 1–2 points at discharge, indicative of a favorable neurologic outcome (29.1% vs. 10.5%, p < 0.001). After adjusting for potential confounders, TTM was significantly associated with reduced odds of in-hospital death (adjusted odds ratio [OR] 0.34, 95% confidence interval [CI] 0.15–0.72, p = 0.006) and poor neurological outcomes (CPC 3–5; adjusted OR 0.30, 95% CI 0.12–0.72, p = 0.008).

**Conclusions:**

Among patients receiving ECPR, the use of TTM is associated with improved neurological outcomes and increased survival to hospital discharge. Future randomized controlled trials are needed to elucidate the pathophysiology and clinical impact of combining TTM with ECPR.

## Introduction

Targeted temperature management (TTM) has been a cornerstone of post-resuscitation care for patients who have suffered a cardiac arrest for several decades. This therapeutic intervention works by mitigating or even preventing the deleterious effects of hypoxic-ischemic encephalopathy (HIE), thereby playing a crucial role in improving neurologic outcomes following a cardiac arrest. While the optimal temperature target within TTM remains a subject of ongoing research and debate, the 2023 American Heart Association guidelines suggest maintaining a stable temperature within a range of 32°C to 37.5°C during post-arrest temperature control [1]. This flexibility allows clinicians to tailor the intervention to the individual patient’s needs while adhering to best practices in post-cardiac arrest care.

In contrast to the neuroprotective focus of TTM, extracorporeal cardiopulmonary resuscitation (ECPR) is an advanced life-saving modality that provides comprehensive hemodynamic support. ECPR involves the initiation of veno-arterial (V-A) extracorporeal membrane oxygenation (ECMO) in patients experiencing cardiac arrest, effectively taking over the function of the heart and lungs to maintain vital organ perfusion. This technique buys critical time for healthcare providers to diagnose and treat the underlying causes of cardiac arrest, thereby potentially improving survival rates and neurological outcomes in selected patient populations. Existing studies have demonstrated that ECPR may improve survival and neurologic outcomes in selected patients with cardiac arrest [2,3]. However, its integration with TTM remains an area of uncertainty. A small study by Ryu et al. [4] suggested that unintentional hypothermia during ECPR may be associated with poor neurological outcomes. Moreover, a retrospective study by Kim et al. found no benefit of TTM in patients undergoing ECPR, raising questions about the interplay between the two interventions [5].

Given these uncertainties, the present study seeks to investigate whether the combination of TTM and ECPR is associated with improved clinical outcomes compared to either therapy alone. By exploring the potential synergistic effects of these interventions, this research aims to provide insights that could be beneficial in clinical practice and optimize care for patients who suffer from cardiac arrest.

## Methods

### Study design and population

This retrospective chart review examined patients who experienced cardiac arrest and underwent extracorporeal cardiopulmonary resuscitation (ECPR) in a medical center in Taiwan between January 2015 and May 2023. Data were accessed for research purposes on 30/11/2023. The study was approved by the Institutional Review Board of China Medical University Hospital (approval number: CMUH112-REC2–053). The requirement for informed consent was waived by the Research Ethics Committee of China Medical University Hospital due to the retrospective nature of the study, as all patient data were anonymized prior to analysis. All procedures complied with relevant regulations and the Declaration of Helsinki.

### In- and exclusion criteria

We included both inpatients and outpatients who experienced cardiac arrest and subsequently underwent ECPR. This approach reflects real-world clinical practice, as cardiac arrest may occur in either hospital or non-hospital settings, and ECPR is initiated once eligible patients arrive at a medical center capable of providing this intervention. Including both groups allowed for a broader evaluation of patient outcomes under different arrest circumstances.

Patients were eligible if they met the following criteria: (1) age ≥ 18 years, (2) occurrence of cardiac arrest in either inpatient or outpatient settings, and (3) receipt of ECPR as documented in the chart review. Patients were excluded if (1) cardiopulmonary resuscitation (CPR) was performed for more than 60 minutes or (2) critical data related to cardiac arrest or major outcomes were missing, including duration of chest compressions, arrest setting, initial rhythm (shockable vs. non-shockable), or Cerebral Performance Categories (CPC) score at discharge.

### Device, ECMO management, targeted temperature control

#### Initiation and criteria for ECPR.

The decision to initiate ECPR was made collaboratively by the emergency physician and cardiovascular surgeons. Once verified, ECPR initiation was activated by the emergency physician and performed by the cardiovascular surgeons. Although definitive criteria were not formally established, ECPR was typically considered for patients with suspected cardiac-origin arrest (e.g., acute myocardial infarction or pulmonary embolism), who had received bystander CPR promptly after collapse, presented with shockable rhythms, and had an estimated collapse-to-ECMO time of less than 60 minutes.

#### VA-ECMO initiation and procedure.

VA-ECMO was initiated in patients with refractory cardiogenic shock characterized by persistent hypotension (systolic blood pressure < 90 mmHg or mean arterial pressure < 65 mmHg) despite adequate fluid resuscitation and high-dose inotropic or vasopressor support. Additional criteria included clinical signs of end-organ hypoperfusion, such as elevated serum lactate levels, reduced urine output, or altered mental status. The timing of VA-ECMO implementation was determined based on the rapid progression of hemodynamic instability and the failure of other therapeutic interventions to restore adequate circulation.

The peripheral VA-ECMO system included a centrifugal pump, a microporous polypropylene hollow fiber membrane oxygenator, and a heparin-coated circuit (Medtronic Inc., MN, USA). Alternatively, a rotaflow pump with a polycarbonate membrane oxygenator and a bioline-coated circuit (Maquet Inc., Rastatt, Germany) was used as a second-line option if the primary oxygenator failed. We employed a 15–19 French (Fr) arterial cannula with a length of 15–18 cm and a 17–21 Fr venous cannula, with a length of 50–60 cm, both inserted via the femoral vessels using a puncture approach. ECMO catheters were introduced in the intensive care unit (ICU), emergency department, or catheterization laboratories. ECMO management and weaning were performed in accordance with the Extracorporeal Life Support Organization guidelines [6].

Low-dose heparin was continuously administered to maintain an activated clotting time of 160–200 seconds. To prevent inadequate venous drainage, right atrial pressure was maintained at or above 12 mmHg, and blood transfusion was performed to maintain a hemoglobin level above 10 g/dL and a platelet count above 80,000/μL. A distal perfusion catheter was routinely inserted into the superficial femoral artery to prevent distal leg ischemia. The initial ECMO flow rate was 3.0–4.0 L/min to assist in the recovery of peripheral circulatory failure. The ECMO flow rate was adjusted based on indicators of organ perfusion, such as mixed venous oxygen saturation, lactic acid levels, and urinary output. ECMO could be weaned off when the flow rate reached 0.8 L/min, echocardiography indicated heart recovery, and organ perfusion indicators were satisfactory.

#### Temperature management protocols.

Following the establishment of the ECMO system, TTM was implemented. The decision to implement TTM or to provide standard normothermic care was made by the attending ICU team based on contemporary guideline recommendations, the patient’s clinical condition, and operational considerations, rather than a fixed institutional protocol. The targeted temperature was determined at the discretion of the ICU team. TTM was delivered either through external cooling pads (“Medivance”ARCTIC SUN 5000 and Sun Arcticgel Pad, BD Inc.) or internal catheters (Thermogard XP Intravascular Temperature Management System, Zoll Medical Inc.).

According to the American Heart Association (AHA) guidelines, a Class I recommendation is to achieve a target temperature of 32°C-36°C within 6 hours, maintain it for 24 hours before transitioning to the rewarming phase [1]. The rewarm rate was set at 0.1°C to 0.25°C per hour until the temperature reached 36.5°C to 37.5°C. By contrast, the non-TTM group comprised patients managed with standard ECMO care without intentional hypothermia protocols, in which body temperature was generally maintained in the normothermic range (36.5–37.5°C) rather than actively lowered or maintained at a hypothermic target.

### Data collection and definition

Two medical residents and one fellow physician from the cardiothoracic surgery department independently conducted thorough chart reviews and documented relevant data in an encrypted hospital database. We collected demographic information, including age, gender, body mass index (BMI), and comorbidities at admission. Disease severity indicators, such as Acute Physiology and Chronic Health Evaluation II (APACHE II) and Sequential Organ Failure Assessment (SOFA) scores, were calculated using data from the first 24 hours post-admission. Additionally, we recorded characteristics of the cardiac arrest event, including pre-arrest rhythm, inpatient or outpatient status, duration of arrest, and the presence of bystander CPR. Outcome variables included successful weaning from ECMO, duration of ECMO support, and survival to discharge. The primary outcome is survival to hospital discharge, which includes patients who are transferred to rehabilitation or extended care facilities, and those requiring home nursing services. Neurological performance was assessed using the CPC scale, a widely recognized five-point scale [7,8]. A score of 1 or 2 indicated a favorable neurologic outcome, while scores of 3 or 4 indicated a poor neurologic outcome, and 5 indicated brain death [9].

The secondary outcome is survival with a favorable neurological outcome, defined as a CPC score of 1 or 2 (with scores of 3–5 considered unfavorable). Additionally, we have specified that the CPC assessment will be performed at the time of hospital discharge to evaluate the neurological status of survivors. Complications were documented, with major bleeding defined as bleeding necessitating four or more units of packed red blood cell transfusions or procedural interventions.

Patients with missing critical demographic or clinical data were excluded prior to analysis. Variables with more than five missing values were omitted, and all analyses were performed using a complete-case approach without imputation.

### Statistical analysis

We conducted data and graph analysis using R software (version 4.4.0, R Foundation for Statistical Computing) and relevant R packages. Categorical variables were analyzed using the Chi-square test or Fisher’s exact test when any group contained five cases or fewer. For continuous variables, distribution patterns were assessed using histogram visualization and the Shapiro-Wilk test. Normally distributed continuous variables were reported as mean ± standard deviation and analyzed using an independent Student’s t-test. Non-normally distributed variables were reported as median (interquartile range (IQR), 25th–75th percentile) and analyzed using the Mann-Whitney U test. A two-tailed p-value less than 0.05 was considered statistically significant. Logistic regression analyses were conducted to estimate the risks of in-hospital mortality and poor neurological outcomes. Initially, univariate logistic regression was performed to examine the association between individual clinical variables and outcomes. Variables with clinical relevance or statistically significant differences between outcome groups were subsequently included in multivariable logistic regression models to assess independent effects while adjusting for potential confounders. The models focused on in-hospital death and poor neurological function as outcome measures. Covariates included sex, targeted temperature management, acute coronary syndrome, shockable rhythm, duration of cardiac arrest, in-hospital cardiac arrest, Acute Physiology and Chronic Health Evaluation scores (APACHE-II), and platelet count. Adjusted odds ratios (ORs) with 95% confidence intervals (CIs) were calculated to quantify the strength of associations, with a p-value < 0.05 considered statistically significant.

## Results

**Fig 1:**
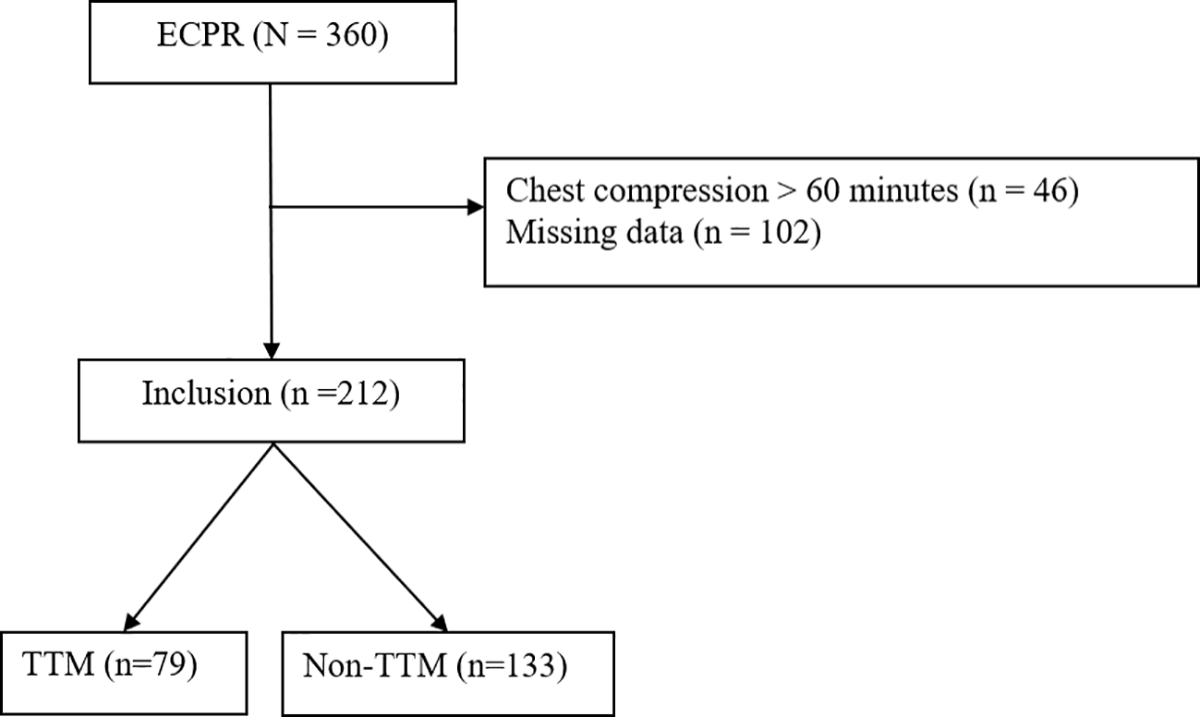
Study flowchart.

A total of 360 adult patients who experienced cardiac arrest and received ECPR were initially screened (Fig 1). Of these, 46 patients were excluded due to prolonged chest compressions exceeding 60 minutes, and 102 patients were excluded due to missing critical data. Consequently, 212 patients were included in the final analysis, with 79 receiving targeted temperature management (TTM) and 133 not receiving TTM.

### Patient characteristics

Patient characteristics were summarized in Table 1. The median age was 59 years (IQR, 49–66), and females comprised 25% of the cohort. There were no significant differences in demographics, such as age and BMI, between the groups, except for gender. Bystander CPR was performed in the majority of cases (83%), with no significant difference observed between the two groups.

**Table 1:** Comparison of patient characteristics between the TTM and non-TTM groups.

Variable	Total (n = 212)	Non-TTM group (n = 133)	TTM group (n = 79)	P Value
Age (years), median (IQR)	59 (49, 66)	60.0 (50.0, 68.0)	56.0 (46.5, 64.5)	0.073
Female, n (%)	54 (25)	46 (34.6)	8 (10.1)	<0.001
Body Mass Index, mean ± S.D.	25.8 ± 4.5	25.4 ± 4.6	26.5 ± 4.3	0.082
Bystander CPR, n (%)	177 (83)	113 (85.0)	64 (81.0)	0.454
In-hospital cardiac arrest, n (%)	120 (57)	91 (68.4)	29 (36.7)	<0.001
Estimated duration of arrest (min)	28 (11, 42)	25.0 (8.0, 40.0)	34.0 (18.5, 47.0)	<0.001
APACHE-II	28.0 (26.0, 32.0)	29.0 (26.0, 33.5)	28.0 (25.5, 30.5)	0.032
SOFA	8.0 (6.0, 11.0)	8.0 (6.0, 11.0)	9.0 (6.5, 11.0)	0.203
VT or VF, n (%)	92 (43)	47 (35.3)	45 (57.0)	0.002
Comorbidities, n (%)				
DM	103 (49)	65 (48.9)	38 (48.1)	0.914
HTN	95 (45)	59 (44.4)	36 (45.6)	0.864
History of CAD	83 (39)	41 (30.8)	42 (53.2)	0.001
Acute coronary syndrome, n (%)	115 (54)	57 (42.9)	58 (73.4)	<0.001
Cause of cardiac arrest, n (%)				<0.001
AMI	126 (59)	61 (45.9)	65 (82.3)	
ARDS	22 (10)	20 (15.0)	2 (2.5)	
Heart failure	7 (3.3)	7 (5.3)	0 (0.0)	
Hypoxemia	1 (0.5)	1 (0.8)	0 (0.0)	
Infective endocarditis	11 (5.2)	9 (6.8)	2 (2.5)	
Pulmonary embolism	15 (7.1)	13 (9.8)	2 (2.5)	
Sepsis	3 (1.4)	3 (2.3)	0 (0.0)	
Trauma	6 (2.8)	2 (1.5)	4 (5.1)	
Unknown	21 (9.9)	17 (12.8)	4 (5.1)	
Laboratory data				
Lactate (mmol/L)	47 (25, 109)	49.7 (23.2, 121.3)	44.6 (28.9, 81.6)	0.579
Platelet count (K/uL)	153 (80, 207)	124.0 (73.3, 189.5)	170.0 (104.5, 223.0)	0.006
Serum creatinine (mg/dL)	1.66 (1.27, 2.75)	1.8 (1.2, 3.0)	1.5 (1.3, 2.2)	0.232
BUN (mg/dL)	25 (19, 42)	28.0 (19.0, 51.0)	24.0 (17.0, 33.0)	0.109
ALT (U/L)	108 (50, 235)	110.0 (45.5, 255.0)	106.5 (61.3, 165.8)	0.92
AST (U/L)	301 (114, 625)	300.0 (112.0, 722.0)	302.0 (114.0, 459.0)	0.56

Abbreviation: TTM, Targeted Temperature Management; CPR, cardiopulmonary resuscitation; APACHE-II, acute physiology and chronic health evaluation II; SOFA, sequential organ failure assessment; VT, ventricular tachycardia; VF, ventricle fibrillation; DM, diabetes mellitus; HTN, hypertension; CAD, coronary artery disease; ARDS, acute respiratory distress syndrome; BUN, blood urea nitrogen; ALT, alanine aminotransferase; AST, Aspartate transaminase.

In-hospital cardiac arrest (IHCA) occurred less frequently in the TTM group compared to the non-TTM group (36.7% vs. 68.4%, p < 0.001). The median duration of cardiac arrest was significantly longer in the TTM group (34 vs. 25 minutes, p < 0.001). The median APACHE-II score was 28 (IQR, 26–32) and the median SOFA score was 8.0 (IQR, 6–11). Shockable rhythms, such as ventricular tachycardia (VT) or ventricular fibrillation (VF), was significantly higher in the TTM group compared to the non-TTM group (57% vs. 35.3%, p = 0.002). The prevalence of comorbidities, including diabetes mellitus, hypertension, and history of coronary artery disease, was similar between the groups. Acute coronary syndrome was more prevalent in the TTM group (73.4% vs. 42.9%, p < 0.001). The leading cause of cardiac arrest was acute myocardial infarction, which was more frequently observed in the TTM group (82.3% vs. 45.9%). Laboratory findings, including lactate levels, serum creatinine levels, and liver function tests, did not show significant differences between the groups. However, platelet counts were significantly lower in the non-TTM group.

### Targeted temperature management, TTM

**Fig 2:**
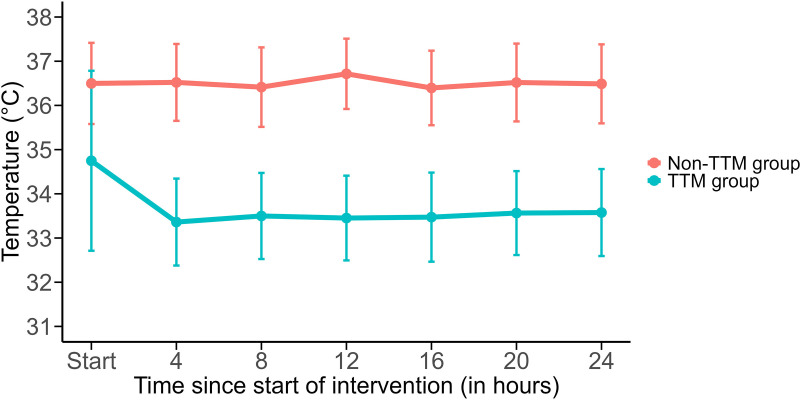
Body temperature in the TTM and non-TTM groups.

S1 Table shows the therapeutic modalities in the TTM group. The methods of TTM delivery included external cooling pads (46%) and internal catheters (54%). The target temperature ranged from 32°C to 36°C, with most patients (66%) had a target temperature set at 33°C. Fig 2 illustrates the evolution of mean body temperature during the first 24 hours after TTM implementation. Outcomes were analyzed by comparing patients who survived to discharge with those who did not (S2 Table). There was no significant difference in the target temperature or methods of TTM delivery between the two groups.

### Complications

S3 Table summarizes the complication rates of the two groups. Overall, 11.4% of patients in the TTM group and 16.5% of patients in the non-TTM group experienced major bleeding (p = 0.305). The non-TTM group had a higher rate of secondary infections, although this did not reach statistical significance (14.3% vs. 8.9%, p = 0.244). There was one case of epilepsy and one case of mesenteric ischemia in the TTM group.

### Comparison of clinical outcomes between TTM and non-TTM groups

In Table 2, patients in the TTM group demonstrated significantly better clinical outcomes compared to the non-TTM group. The rate of successful ECMO weaning was higher in the TTM group (64.6%) than in the non-TTM group (42.1%, p = 0.002). Similarly, survival to hospital discharge was significantly greater in the TTM group (58.2% vs. 38.3%, p = 0.005).

**Table 2:** Comparison of clinical outcomes between the TTM and non-TTM groups.

Variable	Total (n = 212)	Non-TTM group (n = 133)	TTM group (n = 79)	P Value
ECMO weaning, n (%)	107 (50)	56 (42.1)	51 (64.6)	0.002
Survival to discharge	97 (46)	51 (38.3)	46 (58.2)	0.005
Duration of ECMO use (days)	5.0 (3.0, 8.0)	4.0 (3.0, 7.0)	6.0 (5.0, 10.0)	<0.001
Length of hospital stay (days)	8 (4, 30)	5.5 (3.0, 24.0)	11.0 (6.0, 44.5)	<0.001
CPC scores at discharge	4.00 ± 1.31	4.2 ± 1.1	3.6 ± 1.5	0.001
CPC scores at discharge by group, n (%)				<0.001
1 to 2 points	37 (17)	14 (10.5)	23 (29.1)	
3 to 5 points	175 (83)	119 (89.5)	56 (70.9)	
CPC scores at the follow-up outpatient visit	2.62 ± 0.96	2.9 ± 0.9	2.3 ± 1.0	0.002
CPC scores at follow-up outpatient visit (by group), n (%)				0.002
1 to 2 points	37 (44)	13 (28.3)	24 (61.5)	
3 to 5 points	48 (56)	33 (71.7)	15 (38.5)	

Abbreviation: TTM, Targeted Temperature Management; ECMO, Extracorporeal Membrane Oxygenation; CPC, Cerebral Performance Categories.

The duration of ECMO support was longer in the TTM group (median 6.0 days [IQR: 5.0–10.0]) compared to the non-TTM group (4.0 days [IQR: 3.0–7.0], p < 0.001). Hospital length of stay was also significantly longer in the TTM group (11.0 days [IQR: 6.0–44.5]) than in the non-TTM group (5.5 days [IQR: 3.0–24.0], p < 0.001).

Regarding neurological outcomes, the TTM group had lower CPC scores at discharge (mean 3.6 ± 1.5) compared to the non-TTM group (4.2 ± 1.1, p = 0.001), indicating better neurological function. A higher proportion of patients in the TTM group achieved favorable CPC scores (1–2 points) at discharge (29.1% vs. 10.5%, p < 0.001). At follow-up outpatient visits, the mean CPC score remained significantly better in the TTM group (2.3 ± 1.0 vs. 2.9 ± 0.9, p = 0.002), with 61.5% of TTM patients maintaining favorable neurological outcomes (CPC 1–2) compared to 28.3% in the non-TTM group (p = 0.002). Multivariable logistic regression analysis was performed to identify factors independently associated with clinical outcomes, as presented in Table 3. After adjusting for potential confounders, targeted temperature management was significantly associated with lower odds of in-hospital death (adjusted OR 0.34, 95% CI 0.15–0.72, p = 0.006) and poor neurological outcomes (CPC 3–5; adjusted OR 0.30, 95% CI 0.12–0.72, p = 0.008). A longer duration of cardiac arrest was significantly associated with increased odds of in-hospital death (adjusted OR 1.05, 95% CI 1.02–1.07, p < 0.001) and poor neurological outcomes (adjusted OR 1.03, 95% CI 1.01–1.06, p = 0.019). Higher APACHE-II scores were also independently associated with increased odds of both in-hospital death (adjusted OR 1.17, 95% CI 1.08–1.26, p < 0.001) and poor neurological outcomes (adjusted OR 1.13, 95% CI 1.04–1.24, p = 0.006). Additionally, lower platelet counts were significantly associated with in-hospital death (adjusted OR 0.99, 95% CI 0.99–1.00, p = 0.002), but not with poor neurological outcomes. In contrast, acute coronary syndrome and shockable rhythms (VT or VF) were not significantly associated with either outcome.

**Table 3:** Multivariable logistic regression analysis of factors associated with in-hospital mortality and poor neurological outcome.

	In-hospital death			Poor neurologic outcome (CPC 3–5)		
Variable	Adjusted Odds ratio	95% CI	P Value	Adjusted Odds ratio	95% CI	P Value
Sex (female)	1.83	0.78, 4.43	0.171	0.99	0.32, 2.92	0.983
Targeted temperature management	0.34	0.15, 0.72	**0.006**	0.3	0.12, 0.72	**0.008**
Acute coronary syndrome	1.28	0.62, 2.71	0.509	1.46	0.61, 3.53	0.393
Shockable rhythm	0.8	0.40, 1.62	0.536	0.85	0.37, 1.95	0.696
Duration of cardiac arrest	1.05	1.02, 1.07	**<0.001**	1.03	1.01, 1.06	**0.019**
In-hospital cardiac arrest	1.83	0.89, 3.80	0.102	2.06	0.88, 4.93	0.097
APACHE-II scores	1.17	1.08, 1.26	**<0.001**	1.13	1.04, 1.24	**0.006**
Platelet count	0.99	0.99, 1.00	**0.002**	1	0.99, 1.00	0.422

Abbreviation: CPC, Cerebral Performance Categories; APACHE-II, Acute Physiology and Chronic Health Evaluation scores; CI, Confidence Interval.

## Discussion

In summary, this study suggests that among patients undergoing ECPR, targeted temperature management may be associated with improved clinical outcomes, including higher survival-to-discharge rates and better neurological function, even after accounting for confounding factors.

Although patients in the TTM group more frequently presented with acute coronary syndrome and shockable initial rhythms, controlling for these factors did not materially alter the observed association, indicating that the potential benefit of TTM was not solely attributable to arrest etiology or initial rhythm. The absence of significant subgroup effects may reflect limited statistical power and the complex clinical context inherent to ECPR populations. Despite extensive research into the pathophysiology of brain injury following cardiac arrest, there is a lack of studies addressing the physiologic changes in brain circulation and metabolism in the context of ECPR. Animal models in mammals suggest the ECPR in HIE may offer clinical benefits by correcting prolonged low-flow states, which lead to cellular hypoxia and tissue hypoperfusion [10,11]. Rozencwajg et al. [10] induced severe cardiorespiratory failure in sheep and placed them on low-flow (2.5 L min^−1^) versus high-flow (4.5 L min^−1^) V-A ECMO. The sheep in the high-flow group showed improved brain tissue oxygenation and less neuronal shrinkage on brain biopsy. Similarly, Putzer et al. [11] demonstrated that restoring mean arterial pressure to 60 mmHg eight minutes following cardiac arrest improved regional cerebral flow and mitigated cerebral anerobic metabolism. However, it remains unclear whether ECPR can mitigate HIE. Furthermore, while no studies have evaluated the cerebral autoregulation in the context of ECPR, a systematic review [12] suggested that cardiopulmonary bypass may impair cerebral autoregulation by limiting endothelial nitric oxide production or reducing vasoreactivity due to non-pulsatile flow [13]. Additionally, patients undergoing ECPR typically experience prolonged cardiac arrest, which results in extended cerebral ischemia and a low-flow state [14]. While ECPR can rapidly restore the cerebral perfusion, the sudden change in cerebral blood flow may exacerbate the reperfusion injury [15], particularity when accompanied by hyperoxia [16] or hypocapnia [17].

Evidently, ECPR has shown promise in improving outcomes following cardiac arrest. A recent meta-analysis demonstrated that ECPR reduced mortality and improved long-term neurological outcomes, particularly in patients with IHCA [18]. Current literature reports that the rate of favorable neurologic outcomes in patients who received ECPR, defined as CPC 1–2, ranges from 20% to 30% for IHCA and 9% to 41.9% for OHCA [19]. Survival-to-discharge rates vary widely, ranging from 8.4% to 43%. Compared to previous studies, our study shows a similar or slightly higher survival-to-discharge rate (48.8%) but a somewhat lower rate of favorable neurological outcomes at 20.7%, primarily driven by those in the non-TTM groups. This discrepancy may be attributed to variations in ECPR selection criteria and cultural aspects related to end-of-life care.

Our study aligns with the existing literature demonstrating that a shorter duration of CPR [20] is associated with higher survival rates. To focus on patients with a better prognosis, we excluded those who received CPR for more than 60 minutes. This approach is supported by previous studies suggesting that applying stringent criteria, such as age ≤ 65 years, witnessed cardiac arrest with bystander CPR, absence of major comorbidities, and ECMO implementation within 1 hour of arrest, could significantly improve survival rates [21]. Additionally, Yu et al. [22] reported that patients older than 75 years or with low-flow times exceeding 60 minutes had a 0% rate of functional recovery, emphasizing the importance of timely intervention.

Interestingly, in contrast to previous studies [18,23,24], we did not find that IHCA or shockable rhythms were associated with improved clinical outcomes after adjusting for multiple variables. The reasons for this discrepancy remain unclear and warrant further investigation. Future research should aim to define optimal time frames and refine selection criteria, including specific cardiac rhythms and arrest locations, to improve the effectiveness and outcomes of ECPR implementation.

TTM has been shown to improve neurological outcomes in patients experiencing cardiac arrest. The improvement is likely achieved through several mechanisms, including lowering metabolic rate to balance oxygen supply and demand, reducing the levels of excitatory neurotransmitters, preventing ATP depletion, and mitigating cellular apoptosis [25]. There is interest in exploring whether the combination of TTM and ECPR elicits synergistic effects that further improves clinical outcomes. To our knowledge, no randomized controlled trials have specifically addressed this topic. Huang et al. [26] conducted a meta-analysis of prospective observational studies, case series and retrospective cohort studies, which indicated that survival rates and neurological outcomes were not significantly different between patients who received TTM and those who did not. However, many of the included studies were not designed to evaluate the impact of TTM; rather, studies were selected If TTM information was reported for more than 80% of the patient population.

Kim et al. [5] conducted a study with a design similar to ours but found no. benefit of TTM in patients undergoing ECPR. Despite a comparable proportion of patients in the TTM group having IHCA (36.7% in our study versus 32% in Kim et al. study), a higher percentage of patients in the non-TTM group in Kim et al. study had IHCA compared to ours (93.4% versus 68.4%). Notably, the rate of bystander CPR was 97% in the Kim et al. study (100% in the non-TTM group), compared to 83% in our study. This discrepancy may explain why, despite similar rates of favorable neurological outcomes at discharge in the TTM groups (29.1% in our study versus 32% in Kim et al. study), we observed a significantly lower percentage of favorable neurologic outcomes in the non-TTM group compared to Kim et al. study (10.5% versus 34.2%). Moreover, there were notable differences in baseline characteristics between the two studies. For instance, our study had a significantly smaller proportion of female patients (25% versus 31.7% in Kim et al. study), and the median duration of cardiac arrest was shorter in our cohort (28 minutes vs. 44.6 minutes in Kim et al. study). These variations in baseline characteristics may also contribute to the differing outcomes observed between the two studies. TTM could theoretically increase the risks of common complications associated with ECPR, such as bleeding due to hypothermia-induced coagulopathy and secondary infections from hypothermia-induced immunosuppressive effect [27]. However, our study did not reveal a statistically significant difference in complication rates between the non-TTM and TTM groups. Future research is needed to evaluate the impact of TTM on complication rates associated with ECPR.

The present study contributes to the limited body of research evaluating the impact of TTM in patients undergoing ECPR, offering additional data and insights beyond those presented in previous studies. We employed multivariable analysis to control for confounding factors, aiming to minimize bias and provide a more accurate assessment of the effect of TTM. Nonetheless, this study has several limitations. First, the retrospective design limits our ability to establish causality. Second, 102 patients were excluded during the initial screening due to missing data, potentially introducing sampling bias. Third, the unequal gender distribution and limited number of female patients may have reduced statistical power and precision, potentially masking sex-specific differences in outcomes. Fourth, while hypothermia was maintained for 24 hours in the TTM group, actual temperature differences between patients who received TTM and those who did not were not measured. Fifth, more than two-thirds of patients in the TTM group in our study had a target temperature of 33°C. Although the TTM-2 trial demonstrated no mortality or neurological benefit of targeted hypothermia compared with normothermia after OHCA [28], its findings are not directly applicable to our study. TTM-2 exclusively enrolled OHCA patients treated with conventional CPR, whereas our cohort included both in-hospital and out-of-hospital cardiac arrest patients undergoing ECPR. In addition, TTM-2 compared strict hypothermia (33 °C) with targeted normothermia and early fever prevention (body temperature ≥37.8 °C), while in our study, TTM was initiated after ECMO establishment, with target temperatures ranging from 32 to 36 °C in accordance with AHA recommendations. The distinct physiological and hemodynamic context of ECPR may alter the effects of temperature management, highlighting the need for ECPR-specific randomized controlled trials to determine optimal temperature targets and timing. Future trials should also account for the additional logistical complexity and resource demands associated with implementing TTM in conjunction with ECPR.

Additionally, our population included patients with diverse cardiac arrest etiologies, such as trauma, hypoxemia, sepsis, and unknown causes, introducing heterogeneity and limiting the generalizability of the findings. Lastly, CPC scores at follow-up may be limited in accuracy, as only 85 patients completed outpatient follow-up visits. Despite these limitations, both ECPR and TTM remain promising emergent therapies. To validate our findings, future studies should focus on more homogeneous populations and employ prospective designs to better evaluate the effects of TTM and ECPR in specific subgroups, focusing on factors directly assessed in this and similar studies.

## Conclusion

In patients undergoing ECPR, targeted temperature management is associated with a higher likelihood of favorable neurological outcomes and survival to discharge. Future randomized controlled trials are needed to elucidate the pathophysiology and clinical impact of combining TTM with ECPR.

**Key Points**

Question: Is the combination of TTM and ECPR associated with better clinical outcomes compared to either therapy alone?

Findings: Patients in the TTM group had higher rates of successful ECMO weaning, better survival to discharge, and improved neurological outcomes, as evidenced by lower CPC scores. After adjusting for confounders, TTM was associated with lower odds of in-hospital death and poor neurological outcomes.

Meaning: In patients undergoing ECPR, TTM is associated with a higher likelihood of favorable neurological outcomes and survival to discharge.

## Supporting information

S1 Table Therapeutic modalities of targeted temperature management. (DOCX)

S2 Table Factors associated with clinical outcomes of targeted temperature management. (DOCX)

S3 Table Complications. (DOCX)
