# Supplementary material for: Effect of targeted temperature management on neurological and survival outcomes in patients undergoing extracorporeal cardiopulmonary resuscitation
Source: PLoS One. 2026 Feb 10;21(2):e0342473. doi: 10.1371/journal.pone.0342473 (PMC12890137; doi:10.1371/journal.pone.0342473)
Supplement: S1 Table — (DOCX) [file pone.0342473.s001.docx]

Supplementary material

S1 Table. Therapeutic modalities of targeted temperature management

| **Methods of targeted temperature management** | **N (%)** |
| --- | --- |
| \| External pads \| \| --- \| | 36 (46) |
| \| Internal catheters \| \| --- \| | 43 (54) |
| **Target temperature (°C)** | **°C (%)** |
| \| 32 \| \| --- \| | 3 (3.8) |
| \| 33 \| \| --- \| | 52 (66) |
| \| 34 \| \| --- \| | 8 (10) |
| \| 35 \| \| --- \| | 13 (16) |
| \| 36 \| \| --- \| | 3 (3.8) |
|  |  |
