# Supplementary material for: Effect of targeted temperature management on neurological and survival outcomes in patients undergoing extracorporeal cardiopulmonary resuscitation
Source: PLoS One. 2026 Feb 10;21(2):e0342473. doi: 10.1371/journal.pone.0342473 (PMC12890137; doi:10.1371/journal.pone.0342473)
Supplement: S2 Table — (DOCX) [file pone.0342473.s002.docx]

Supplementary material

S2 Table. Factors associated with clinical outcomes of targeted temperature management

|  | Deceased | Survival to discharge | P-value |
| --- | --- | --- | --- |
| Target temperature (°C) | 33.0 (33.0, 35.0) | 33.0 (33.0, 34.0) | 0.419 |
| Number of patients who received TTM through the internal catheter | 17 (51.5%) | 26 (56.5%) | 0.659 |

Abbreviation: TTM, Targeted Temperature Management.
