# Supplementary material for: Effect of targeted temperature management on neurological and survival outcomes in patients undergoing extracorporeal cardiopulmonary resuscitation
Source: PLoS One. 2026 Feb 10;21(2):e0342473. doi: 10.1371/journal.pone.0342473 (PMC12890137; doi:10.1371/journal.pone.0342473)
Supplement: S3 Table — (DOCX) [file pone.0342473.s003.docx]

Supplementary material

S3 Table. Complications

| **Variables** | **Total**  **n = 212 (%)** | **Non-TTM group**  **n = 133 (%)** | **TTM group**  **n = 79 (%)** | **P Value** |
| --- | --- | --- | --- | --- |
| Major bleeding | 31 (15) | 22 (16.5) | 9 (11.4) | 0.305 |
| Gastrointestinal bleeding | 16 (52) | 13 (59.1) | 3 (33.3) |  |
| Intracranial bleeding | 11 (35) | 5 (22.7) | 6 (66.7) |  |
| Mediastinum bleeding | 1 (3.2) | 1 (4.5) | 0 (0.0) |  |
| Unknown site | 3 (9.7) | 3 (13.6) | 0 (0.0) |  |
| Secondary infection | 26 (12) | 19 (14.3) | 7 (8.9) | 0.244 |
| Bacteremia | 19 (73) | 16 (84.2) | 3 (42.9) |  |
| Pneumonia | 2 (7.7) | 0 (0.0) | 2 (28.6) |  |
| Sepsis | 1 (3.8) | 0 (0.0) | 1 (14.3) |  |
| Unknown | 4 (15) | 3 (15.8) | 1 (14.3) |  |
| Others | 16 (7.5) | 7 (5.3) | 9 (11.4) | 0.102 |
| Epilepsy | 1 (6.3) | 0 (0.0) | 1 (11.1) |  |
| Hypoxic encephalopathy | 5 (31) | 1 (14.3) | 4 (44.4) |  |
| Mesenteric ischemia | 5 (31) | 4 (57.1) | 1 (11.1) |  |
| Sepsis | 3 (19) | 1 (14.3) | 2 (22.2) |  |
| Unknow | 2 (13) | 1 (14.3) | 1 (11.1) |  |
